# Supplementary material for: Deletion of wfs1 Impairs Oligodendrocyte Precursor Cells Dorsal Distribution and Myelination Through the wfs1-hmgcs1 Axis in Zebrafish
Source: Biology (Basel). 2026 Aug 16;15(16):1402. doi: 10.3390/biology15161402 (PMC13510151; doi:10.3390/biology15161402)
Supplement: Supplementary file 1 [file biology-15-01402-s001.zip › File S1-Transcriptome sequencing data and differentially expressed genes (DEGs) in wfs1-- zebrafish at 5 dpf/1.GO/wfs1KOvsWT/GSEA/WT/REGULATION_OF_DNA_REPLICATION/REGULATION_OF_DNA_REPLICATION(GO_0006275).html]

Details for gene set REGULATION\_OF\_DNA\_REPLICATION(GO:0006275)[GSEA]


|  || Dataset | deg.sample |
| Phenotype | sample.cls |
| Upregulated in class | WT |
| GeneSet | REGULATION\_OF\_DNA\_REPLICATION(GO:0006275) |
| Enrichment Score (ES) | -0.5514162 |
| Normalized Enrichment Score (NES) | -1.96208 |
| Nominal p-value | 0.0 |
| FDR q-value | 0.14532468 |
| FWER p-Value | 0.261 |
Table: GSEA Results Summary

  

Fig 1: Enrichment plot: REGULATION\_OF\_DNA\_REPLICATION(GO:0006275)
  
Profile of the Running ES Score & Positions of GeneSet Members on the Rank Ordered List

  

| PROBE | DESCRIPTION   (from dataset) | GENE SYMBOL | GENE\_TITLE | RANK IN GENE LIST | RANK METRIC SCORE | RUNNING ES | CORE ENRICHMENT || 1 | 566954 | terf1 |  |  | 5522 | 0.634 | -0.1308 | No |
| 2 | 565952 | e2f8 |  |  | 5916 | 0.596 | -0.1032 | No |
| 3 | 445395 | esco2 |  |  | 7438 | 0.458 | -0.1202 | No |
| 4 | 100333282 | esco1 |  |  | 7957 | 0.426 | -0.1079 | No |
| 5 | 100148564 | gmnc |  |  | 8431 | 0.408 | -0.0954 | No |
| 6 | 447872 | dscc1 |  |  | 11584 | 0.212 | -0.1801 | No |
| 7 | 445819 | wdr18 |  |  | 13179 | 0.131 | -0.2213 | No |
| 8 | 566753 | dbf4b |  |  | 13517 | 0.115 | -0.2242 | No |
| 9 | 566787 | cdt1 |  |  | 13621 | 0.110 | -0.2200 | No |
| 10 | 567941 | e2f7 |  |  | 14812 | 0.055 | -0.2537 | No |
| 11 | 553237 | zranb3 |  |  | 14888 | 0.052 | -0.2526 | No |
| 12 | 798920 | igfbp2b |  |  | 15624 | 0.016 | -0.2746 | No |
| 13 | 393967 | pds5a |  |  | 15806 | 0.006 | -0.2799 | No |
| 14 | 445411 | ticrr |  |  | 16257 | -0.009 | -0.2933 | No |
| 15 | 799320 | znf365 |  |  | 18613 | -0.104 | -0.3603 | No |
| 16 | 30678 | pcna |  |  | 18983 | -0.121 | -0.3638 | No |
| 17 | 560412 | smarcal1 |  |  | 20496 | -0.188 | -0.3986 | No |
| 18 | 555786 | npm2b |  |  | 20606 | -0.194 | -0.3890 | No |
| 19 | 777638 | ctc1 |  |  | 21066 | -0.219 | -0.3887 | No |
| 20 | 325855 | timeless |  |  | 21274 | -0.229 | -0.3799 | No |
| 21 | 445058 | tipin |  |  | 26741 | -0.603 | -0.5110 | Yes |
| 22 | 559535 | dna2 |  |  | 27848 | -0.722 | -0.4973 | Yes |
| 23 | 402963 | zgc:77398 |  |  | 28685 | -0.854 | -0.4662 | Yes |
| 24 | 799015 | dbf4 |  |  | 29656 | -1.114 | -0.4219 | Yes |
| 25 | 368320 | gmnn |  |  | 31570 | -3.128 | -0.2721 | Yes |
| 26 | 794176 | igfbp2a |  |  | 31758 | -4.206 | 0.0041 | Yes |
Table: GSEA details
[plain text format]

  

Fig 2: REGULATION\_OF\_DNA\_REPLICATION(GO:0006275)
  
Blue-Pink O' Gram in the Space of the Analyzed GeneSet

  

Fig 3: REGULATION\_OF\_DNA\_REPLICATION(GO:0006275): Random ES distribution
  
Gene set null distribution of ES for
 **REGULATION\_OF\_DNA\_REPLICATION(GO:0006275)**
